# Identification of Characteristic Autoantibodies Associated With Deficiency Pattern in Traditional Chinese Medicine of Rheumatoid Arthritis Using Protein Chips

**DOI:** 10.3389/fphar.2019.00755

**Published:** 2019-07-10

**Authors:** Heru Zhao, Yin Zhang, Bin Liu, Li Li, Lulu Zhang, Mei Bao, Hongtao Guo, Haiyu Xu, Hui Feng, Lianbo Xiao, Wenjun Yi, Jianfeng Yi, Peng Chen, Cheng Lu, Aiping Lu

**Affiliations:** ^1^Key Laboratory for Research on Active Ingredients in Natural Medicine of Jiangxi Province, Yichun University, Yichun, China; ^2^Institute of Basic Research in Clinical Medicine, China Academy of Chinese Medical Sciences, Beijing, China; ^3^Department of Rheumatology, First Affiliated Hospital of Henan University of TCM, Zhengzhou, China; ^4^Institute of Chinese Materia Medica, China Academy of Chinese Medical Sciences, Beijing, China; ^5^Shanghai Guanghua Hospital of Integrated Traditional Chinese and Western Medicine, Shanghai, China; ^6^China Association of Acupunture and Moxibustion, Beijing, China; ^7^Beijing Key Laboratory of Traditional Chinese Medicine Basic Research on Prevention and Treatment for Major Diseases, Experimental Research Center, China Academy of Chinese Medical Sciences, Beijing, China; ^8^Law Sau Fai Institute for Advancing Translational Medicine in Bone & Joint Diseases, School of Chinese Medicine, Hong Kong Baptist University, Kowloon Tong, Hong Kong

**Keywords:** autoimmune disease, rheumatoid arthritis, deficiency pattern, autoantibody, protein chips, vascular endothelial growth factor A165

## Abstract

**Background:** Rheumatoid arthritis (RA) is an autoimmune disease. Based on traditional Chinese medicine (TCM) theory, deficiency pattern (DP) which leads to specific treatment principles in clinical management is a crucial pattern diagnosis among RA patients, and autoantibodies have potential implications in TCM pattern classification. The purpose of this study was to identify specific RA DP-associated autoantibodies.

**Methods:** RA DP patients, RA nondeficiency pattern (NDP) patients and healthy controls (HCs) were recruited for this study. Then, clinical data and sera from all subjects were collected. After that, the sera were probed with protein chips, which were constructed by known RA related autoantigens, to screen for DP-associated candidate autoantibodies. Lastly, candidate autoantibodies were validated *via* enzyme-linked immunosorbent assay (ELISA) and function was evaluated by network analysis.

**Results:** Protein chips results showed that RA patients have higher levels of anti-vascular endothelial growth factor (VEGF) A165 antibodies than HC (*P* < 0.005); anti-VEGFA165 antibodies levels of patients with RA DP were lower than patients with RA NDP (*P* < 0.05). The results of the ELISA also showed statistically significant differences in anti-VEGFA165 antibodies between the RA and HC group (*P* < 0.0001); and there were statistically significant differences in anti-VEGFA165 antibodies between the RA DP and RA NDP group (*P* < 0.05). Network analysis results suggested IL-6 signaling pathway has a significant effect on VEGFA165 in RA patients.

**Conclusion:** Autoantibodies identification in RA using protein chips help in understanding DP in TCM. Discovery of anti-VEGFA165 antibodies may provide the possibility for clinical precision treatment.

## Introduction

Rheumatoid arthritis (RA) is a common autoimmune disease, accounting for more than 1% of the world’s population (Uhlig et al., 2014). Although the exact cause of RA is still unclear, immunity disorder and chronic inflammation are generally considered as contribution to RA (Okada et al., 2010). In the later stages, other parts/organs of the RA patients may be affected, resulting in disability, declined quality of life, decreased ability to work (de Hair et al., 2013), and improved health care utilization (Boonen and Severens, 2011). In recent years, research has focused on the disease in the field of RA, leading to the discovery that autoantibodies can precede the clinical onset of the disease by many years (van Boekel et al., 2002).

Traditional Chinese medicine (TCM) techniques have shown therapeutic promise in treating RA (Goldbach-Mansky et al., 2009). In TCM theory, RA is considered an impediment disease (“Bi” pattern in Mandarin) and is caused by the invasion of heat pathogens, wind, or dampness into the human body (Li, 2002). According to RA patients symptoms further stratified, a pattern diagnosis can determined for a subgroup of the patients, and then will prescribe specific therapy based on these pattern classifications, the special treatment principle can improve clinical efficacy (Lu et al., 2012). Deficiency pattern (DP) as key pattern diagnosis among RA patients can lead to a specific treatment principle of “tonifying the DP” in clinical management (Wang et al., 2013). In the practice of TCM, it have been found that Tripterygium glycosides and qingluo tongbi granules are safe and effective in the treatment of RA DP (Zhou et al., 2004), thus the treatment of RA DP has a good efficacy.

Autoantibodies can distinguish subgroups of the autoimmunity disease. Such as adult latent autoimmune diabetes and phenotypic type 2 diabetes can be distinguished by autoantibodies against zinc transporter 8, insulin, insulinoma antigen-2 and glutamic acid decarboxylase (Sorgjerd, 2018). There are two types of autoimmune hepatitis: type 1 (smooth muscle antibodies and antinuclear antibodies) and type 2 (liver cytosol antibodies and/or antibodies to liver and kidney microsomes type 1 and/or antibodies to liver and kidney microsomes type 3), which can be classified based on autoantibodies (Zhang et al., 2014). Some scholars found that the positive rates of anti-U1 RNP antibodies were significantly different between the systemic lupus erythematosus DP and systemic lupus erythematosus TCM excess pattern groups (Dai et al., 2016). It was discovered that a significant difference exists between antinuclear antibodies in the kidney DP and kidney excess pattern groups (Guo et al., 2013). Autoantibodies have potential implications in TCM RA pattern classification.

The advent of protein chip technology has enabled a large-scale analysis of proteins to recognize biomarkers of RA subtypes, and identify the mechanisms underlying these subtypes (Hueber et al., 2005). We applied contain panels of RA autoantigens arrays to profile autoantibodies in sera derived from RA DP patients, identified autoantibodies associated with RA DP, and further validated protein chip results by ELISA. Finally, we analyzed the possible network of the association between RA DP and autoantibodies.

## Methods

### Serum Samples

All the samples were collected from female RA patients (patients with RA include RA DP and non-deficiency RA) in Henan Province Hospital of TCM, included 20 sera from RA DP, 40 sera from RA NDP (nondeficiency pattern), and 40 HC matched by age and gender. Of which 15 sera of RA DP, 15 sera of RA NDP, and 15 sera of HC were used for screening of protein chips, and all the samples were used for ELISA validation. RA patients were eligible to participate if they had met the American College of Rheumatology criteria for RA for at least one year, with functional classes of I, II, or III (Arnett et al., 1988). Weakness in the waist, fatigue, dizziness, heavy limbs, nocturia, and numb limbs (Zhang et al., 2012), inhibited stretching and bending in limbs, pain occurring or worsening during moodiness, pain occurring or worsening at night, and deformity were categorized in TCM RA DP (Wang et al., 2013). NDP means that there is no typical DP in the RA. Patients with RA DP were recorded with whole clinical manifestations according to TCM theory using a questionnaire, a tongue examination, and pulse diagnosis by 3 appointed TCM practitioners. Patients were included in the study only if the 3 practitioners reported consistent results. This study was approved by the Ethics Committee at the Institute of Basic Research in Clinical Medicine, China Academy of Chinese Medical Sciences, and was conducted according to the standards of the Declaration of Helsinki. Written informed consent was obtained from the participants.

### Gene Cloning, Expression, Purification, and Mass Spectrometry

We expressed and purified seven proteins. Extraction of RNA from cells was carried out as per TRIzol reagent kit instructions (Invitrogen, CA; Lot No. 291946AX). Then amplification of genes was performed by RT-PCR using kit instructions (Biomake, Shanghai, China; Lot No: 20). The PCR product of the gene was sequenced by Sangon Biology (Shanghai, China). Proteins were over expressed in pET28a and then purified the recombinant protein by Ni-NTA kit (CWBIO, Beijing, China; Lot No.01376/20302). Purified recombinant proteins were confirmed by proteomics analyzer AB 4700 mass spectrometry (Applied Biosystems, Foster City, CA).

### Fabrication of the Protein Chips

Thirty autoantibodies associated with RA were found by reading the literature, and then designed a protein matrix using these autoantibodies. Printed autoantigens included 22 purchased, 7 expressed proteins (SinoBiological, Beijing, China; Diarect AG, Freiburg, Germany; Arotec, São Paulo, Brazil), and 1 protein extract that was derived from human stratum corneum whole protein. A 12-hole rubber gasket (CapitalBio, Beijing, China; Lot No.0011016) was applied to each chip to form 12 individual chambers. All the proteins were printed in duplicate within chamber, and identical probe areas were fabricated in 12 chambers. Each chamber included positive spots (human IgG) and blank spots. The printed chips were allowed to remain at room temperature for 1 h before storage at 4°C.

### Serum Assays on Protein Chips

Blocked 1 h with PBS containing 5% fetal calf serum (Lot No. 18040502), incubated 2 h with 40 μl of 1:50 dilutions of RA DP patients, RA NDP patients and HC sera, and washed three times with PBST. Then incubated with a 1:400 dilution of Cy3-conjugated goat anti-human IgG secondary antibody (Lot No. AA03187869) and followed by PBST washing three times. The chips were scanned using a microarray scanner (CapitalBio, Beijing, China).

### ELISA

The candidate proteins were coated onto 96-well plates and overnight at 4°C. The CB coating solution was prepared as follows: 0.795 g of Na_2_CO_3_ and 1.465 g of NaHCO_3_ were dissolved in 500 ml of water and stored at 4° C. 200 μl of 5% fetal calf serum (Lot No. 18040502) was added to each well for blocked nonspecific binding. 100 μl human serum (1:50) was added to each well and incubated for 75 min at 37°C, then the plates were washed three times with PBST. 100 μl of horseradish peroxidase-labeled mouse anti-human IgG monoclonal antibody was added (1:10,000; Beijing Protein Innovation Co. Ltd; 130499) to each well for 45 min and then we washed the plates three times with PBST. 50 μl of tetramethylbenzidine substrate solution was added (makewoderbio, Beijng, China; Lot No.20180813). After this the plates were kept in a dark, room-temperature place for 8 min. The reaction was stopped after 50 μl H_2_SO_4_ entry and the immunoreactivity was measured by reading A450 (BioTek, Winooski, Vermont).

### Statistical Analysis and Network Analysis

Chemiluminescent signals were acquired using microarray scanner, and the intensity value (signal intensity − background) was normalized using the mean of positive point signals. Each protein is in duplicate print, the average of each replicate is used as the final signal intensity for a given protein. Using a value of two times the standard deviation above mean_healthy_ as a cutoff value. SAS version 9.4 (SAS Institute Inc, *LICENSE = SAS 000062456227) was used to perform means, standard deviation and the t-test, *P*-value (*P* < 0.05) was taken as significant. Network analysis was conducted using the Ingenuity Pathway Analysis system (IPA, Ingenuity^®^ Systems, http://www.ingenuity.com).

## Results

### Baseline Characteristics of Study Subjects

The characteristics of the enrolled subjects, including age, ESR, CRP, RF-IgG, RF-IgA, RF-IgM, complement C3, complement C4, anti-dsDNA, anti-CCP were shown in **Table 1**. The age of the RA DP patients was older than that of the RA NDP patients (*P* < 0.0005). There was no significant difference in other indicators between the RA DP and RA NDP patients. Two groups used for protein chips technology or ELISA showed the above results.

**Table 1:** Characteristics among the groups of enrolled subjects.

Indicators/Groups	DP (protein chip)	NDP (protein chip)	DP (ELISA)	NDP (ELISA)
Number of subjects	15	15	20	40
Age (years)	59.8 ± 8.94*	49.33 ± 9.66	62.32 ± 10.00**	49.72 ± 12.36
Duration of RA (month)	151.45 ± 144.11	120.14 ± 112.21	160.10 ± 160.80	96.72 ± 112.00
ESR (mm/h)	45.53 ± 28.02	57.4 ± 29.35	50.05 ± 29.34	48.21 ± 29.77
CRP (mg/L)	34.04 ± 38.61	39.31 ± 42.45	35.89 ± 37.29	28.78 ± 33.69
RF-IgG (RU/ml)	32.8 ± 73.53	16.92 ± 20.94	28.29 ± 67.53	15.99 ± 23.63
RF-IgA (RU/ml)	112.92 ± 109.71	132.11 ± 114.61	131.43 ± 110.52	124.39 ± 105.95
RF-IgM (RU/ml)	230.93 ± 134.67	301.16 ± 171.55	260.02 ± 140.76	296.92 ± 134.08
Complement C3 (g/L)	1.28 ± 0.24	1.26 ± 0.19	1.27 ± 0.22	1.24 ± 0.17
Complement C4 (g/L)	0.28 ± 0.13	0.26 ± 0.08	0.29 ± 0.12	0.26 ± 0.07
Anti-dsDNA (IU/ml)	3.53 ± 0.80	3.88 ± 0.69	3.34 ± 0.89	3.88 ± 0.76
Anti-CCP (RU/ml)	65.58 ± 42.71	78.09 ± 51.55	71.91 ± 41.50	85.43 ± 46.56

ESR indicates erythrocyte sedimentation rate; CRP, C reactive protein; RF-IgG, rheumatoid factor immunoglobulin G; RF-IgA, rheumatoid factor immunoglobulin A; RF IgM, rheumatoid factor immunoglobulin M; Anti-dsDNA, anti-distrand-DNA antibody; Anti-CCP, anti-cyclic citrullinated peptide.

The comparisons of clinical indicators between DP and NDP. Unpaired t-test was applied to continuous variables analysis, and the data are expressed as the mean ± SD when appropriate (95% CI). The DP (protein chip) groups age vs NDP (protein chip) groups age *P < 0.05, DP (ELISA) groups age vs NDP groups age (ELISA) **P < 0.01.

### Gene Cloning, Expression, and Purification of Proteins

**Figure 1:**
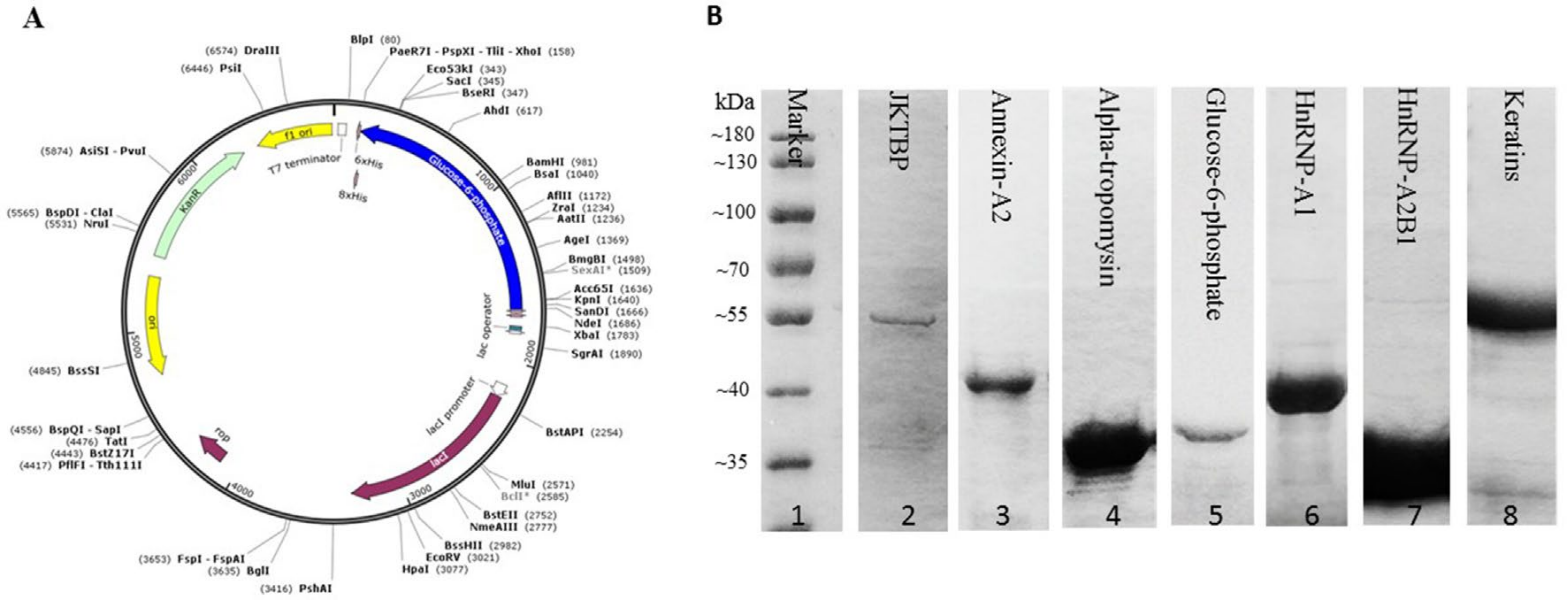
Gene cloning, expression and purification of autoantigens for protein chip preparation. **(A)** Synthetic gene was cloned into NdeI/XhoI digested pET28a. **(B)** Purified proteins were separated with 10% SDS-PAGE and then stained with coomassie brilliant blue. Lane 1: protein marker, Lane 2: JKTBP, Lane 3: Annexin-A2, Lane 4: Alpha-tropomysin, Lane 5: Glucose-6 phosphat, Lane 6: Hnrnp-A1, Lane 7: Hnrnp-A2B1, Lane 8: Keratins isoform of heterogeneous nuclear ribonucleoprotein D-like = JKTBP, heterogeneous nuclear ribonucleoprotein A2B1 = HnRNP A2B1, heterogeneous nuclear ribonucleoprotein A1 = HnRNP A1.

Seven proteins were expressed and purified for fabrication of protein chips. First, synthetic gene was cloned into pET28a (**Figure 1A**). Second, coomassie brilliant blue staining of expressed proteins on SDS-PAGE, purified proteins were separated with 10% SDS-PAGE and then stained with coomassie brilliant blue. Visible proteins migrated at their expected molecular weights (**Figure 1B**). Last, proteins from Ni-NTA resin were reassured by mass spectrometry, and the results show that putative proteins have a highly homologous identity (*P* < 0.05).

### Construction of RA-associated Protein Chips

**Figure 2:**
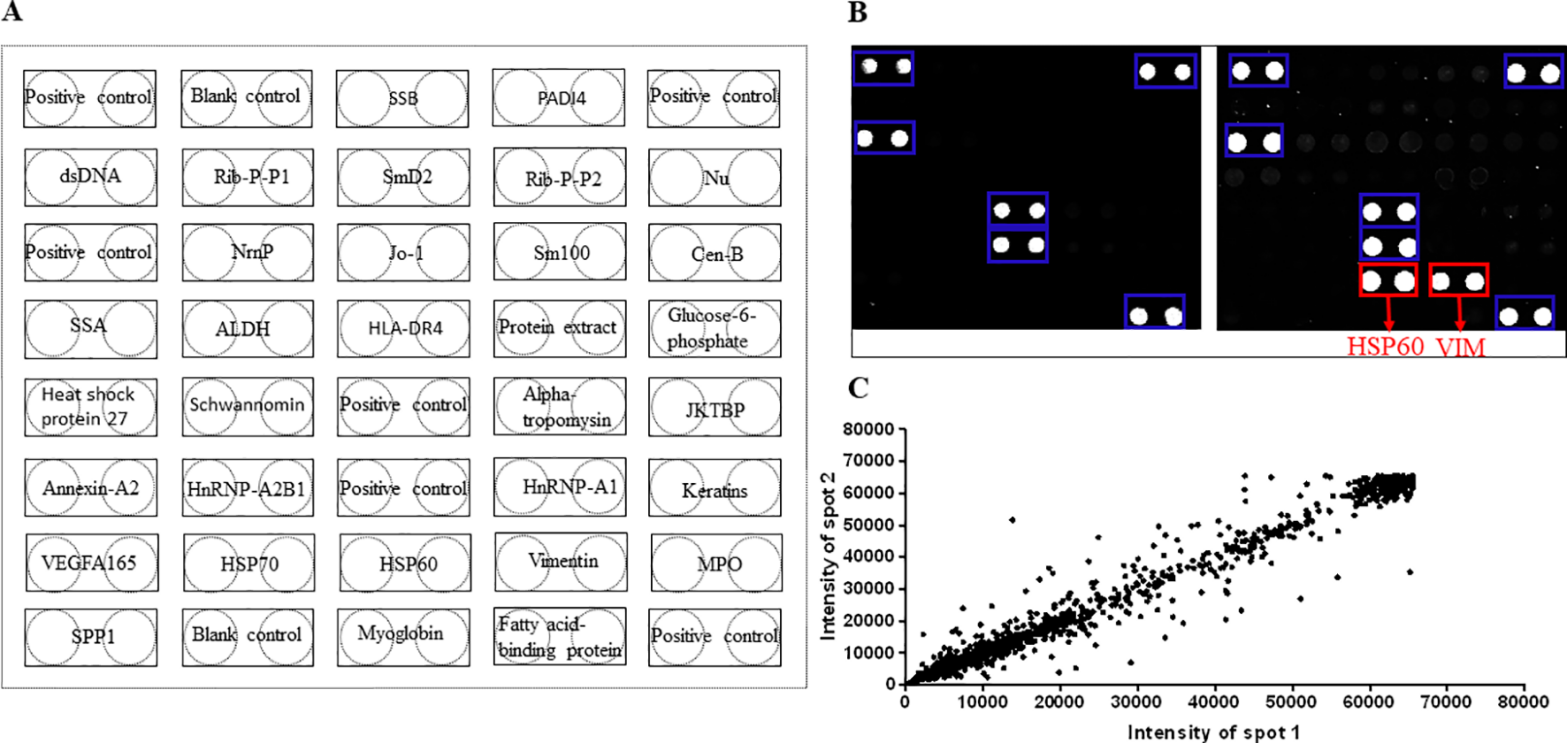
Construction RA associated protein chip. **(A)** Human proteins were purified and printed in duplicate on poly-L-lysine coated microscope slides. **(B)** Correlation of spot intensities of all the duplicate pairs. The signal intensities of duplicate spots (Spot 1 versus its corresponding Spot 2) were plotted against each other. The resulting correlation coefficient was 0.97, indicating high reproducibility of the protein spotting. **(C)** To monitor the quality and relative quantity of the printed proteins on glass slides, the human protein chips were probed with anti-His antibody, followed by Cy3-labeled secondary antibody to visualize the signals, red boxes indicate HIS-tag group. Blue boxes indicate human IgG (the positive control), red boxes indicate HSP60 and VIM antibody. Sjogren syndrome antigen B = SSB, peptidyl arginine deiminase 4 = PADI4, double-stranded DNA = dsDNA, ribosomal protein P1 = Rib-P-P1, smithD2 = SmD2, ribosomal protein P2 = Rib-P-P2, nucleosome = Nu, nuclear ribonucleoprotein = NrnP, smith100 = Sm100, centromere protein b = Cen-B, sjogren syndrome antigen A = SSA, aldehyde dehydrogenase = ALDH, human leukocyte antigen-DR4 = HLA-DR4, isoform of heterogeneous nuclear ribonucleoprotein D-like = JKTBP, heterogeneous nuclear ribonucleoprotein A2B1 = HnRNP A2B1, heterogeneous nuclear ribonucleoprotein A1 = HnRNP A1, vascular endothelial growth factor A165 = VEGFA165, Heat shock protein 70 = HSP70, heat shock protein 60 = HSP60, myeloperoxidase = MPO, osteopontin = SPP1.

In order to optimize the serum profiling assay, we tested a variety of different conditions in a pilot assay. And polymer-slide H was chosen for chips fabrication, 20-fold dilution was chosen as the most appropriate serum concentration, 400-fold dilution was chosen as the most appropriate Cy3-conjugated anti-human IgG antibody. Finally, we employed the protein chips containing 30 RA-associated autoantigens to perform serum profiling of samples collected from 15 DP, 15 NDP, and 15 HC subjects (**Figure 2A**). Human IgG at a known concentration was printed at each chamber on the chips to serve as a control and landmark. Anti-vimentin antibodies and anti-heat shock protein 60 antibodies were incubated in 1 individual chambers for checkout the protein’s activity (**Figure 2B**). The results showed protein chips had a high correlation coefficient (0.978) between duplicate spots, which suggested that it was of high quality (**Figure 2C**).

### Probing Results of the Protein Chips

**Figure 3:**
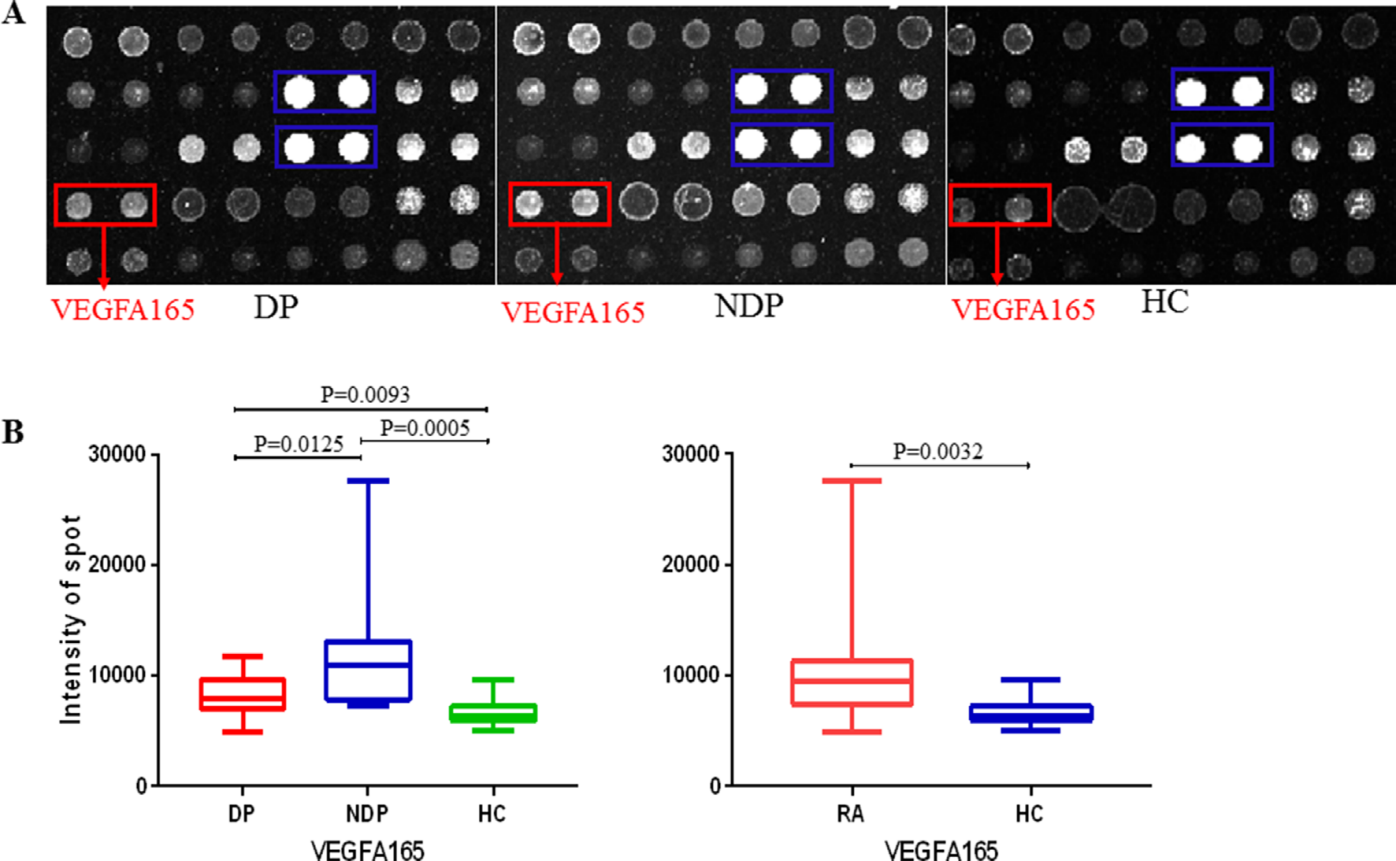
Probing results of the human protein chip with DP and NDP sera. **(A)** Fifteen DP, 15 NDP, and 15 HC serum samples were diluted 1:20 and individually incubated with the human protein chip, followed by the addition of the anti-human IgG antibody (Cy3-conjugated). Chips were dried and scanned to acquire the images. Representative areas of the images are illustrated. Red boxes indicate the positive candidate autoantigens, and blue boxes indicate human IgG (the positive control). **(B)** Fifteen DP, NDP, and 15 HC. The signal distributions of VEGFA165 reacting with the serum samples is displayed. The bar within the rectangle indicates the median value.

We observed that autoantigens could be readily recognized by sera from DP and NDP groups, and the serum profiles also showed obvious individual-to-individual variation within among groups (**Figure 3A**). To identify potential RA DP-associated autoantigens, we used microarray scanner to acquire the resultant signal intensities of all protein spots in each assay and identified the positives within each chip (see details under Methods). Protein chips results showed that RA have higher levels of anti-vascular endothelial growth factor (VEGF) A165 (P15692) antibodies than HC (*P* < 0.005); anti-VEGFA165 antibodies levels of patients with RA DP were lower than patients with RA NDP (*P* < 0.05); patients with RA DP have higher levels of anti-VEGFA165 antibodies than HC (*P* < 0.01). There was no statistical difference between RA DP and RA NDP groups for other autoatibodies. Using a value of two times the standard deviation above mean_healthy_ as a cutoff value, an anti-IgG antibody reaction against VEGFA165 was observed in 2 out of 15 RA DP patients (13%), 6 out of 15 RA NDP patients (40%), 0 out of 15 HC patients (0%). Finally, to visualize the range of signal intensities, we conducted box-whisker plot analysis for anti-VEGFA165 antibodies across the various groups of sera (**Figure 3B**).

### Verification by ELISA

**Figure 4:**
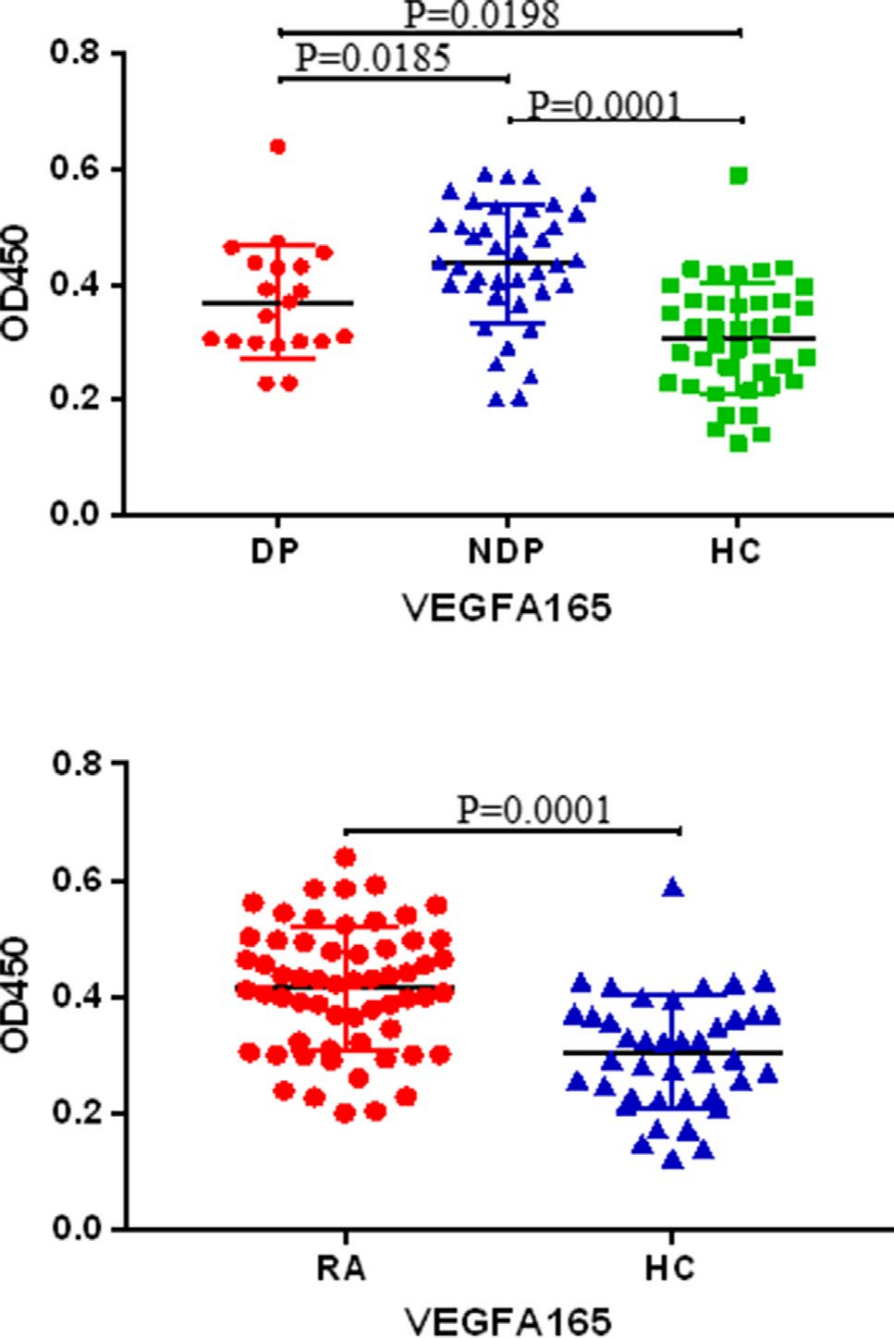
The result of protein chip was verified by enlarging the sample by ELISA. Sera derived from DP (n = 20), NDP (n = 40) and HC (n = 40) were tested for VEGFA165 autoantibody using VEGFA165.

In order to validate the candidate autoantibodies identified using protein chips technology. ELISA of anti-VEGFA165 antibodies were carried out with 20 RA DP, 40 RA NDP, and 40 HC subject sera to verification. The results showed reactivity of RA serum IgG antibodies against were higher than HC (*P* < 0.0001); the reactivity of RA DP serum IgG antibodies against were lower than NDP (*P* < 0.05), and the reactivity of RA DP serum IgG antibodies against were higher than HC (*P* < 0.05) (**Figure 4**). Therefore, the anti-VEGFA165 antibodies were confirmed as being able to distinguish between RA DP and RA NDP.

## Discussion

TCM pattern classification analysis was carried out through data collected by four combined diagnostic methods, including WANG (inspection), WEN (falling-rising tone, auscultation, and olfaction), WEN (falling tone, inquiry), and QIE (palpation) (Tao et al., 2017). The “Cold,” “Heat,” “Deficiency,” and “Excess” were four basic patterns in TCM (Jiang, 2005). Pattern classification was the advantage of TCM, which can guide herbal prescriptions (Chai et al., 2011). Some TCM herbal formulae have been reported for the treatment of DP, such as WuziYanzong Pill, which protected the impaired spermatogenesis possibly by mediating mitochondrial energy metabolism in DP rats (Liu, 2014). Both crude and bran-processed rhizome of Atractylodes lancea have alleviated the symptoms of spleen DP in rats (Xue et al., 2018). Radix Astragali and its split components can promote water metabolism in rats with the syndrome of dampness stagnancy due to spleen deficiency by regulating the expression of AQPs (Zhao et al., 2017). Xiaoyaosan intervention may effectively adjust the gut dysbacteriosis in functional dyspepsia (Qiu et al., 2017). TCM has a good effect in treating DP.

In TCM practice, an experiential diagnosis approach has been frequently used in pattern classification in RA patients (Mo et al., 2016). In order to promote the traditional experiential diagnosis, the scientific evidence for pattern classification is essential, and it would be beneficial to understand essence of the pattern classification. The study suggested that statistical-based clinical data classification had a similar TCM pattern differentiation in RA patients (He et al., 2008). We had previous used integrating liquid chromatography/mass spectrometry and gas chromatography/mass spectrometry platforms in conjunction with the Ingenuity Pathway Analysis software identified the biomarkers in RA patients with typical TCM cold or heat patterns (Gu et al., 2012; Guo et al., 2016). And we identified the network-based gene expression biomarkers for both cold- and heat-patterns of RA by obtained gene expression profilings of CD_4_ + T cells from cold-pattern RA patients and heat-pattern RA patients using microarray (Lu et al., 2012). In this study, we explored the autoantibody of sera in RA patients with typical TCM DP and NDP by protein chips technology, and anti-VEGFA165 antibodies which DP-associated in RA was identified.

Our previous study of the TCM DP-related genes network study found TCM DP is probably related to immune response (Wang et al., 2013). Mo, Na et al. found that the age of disease onset exhibited significant differences between DP and NDP, which is consistent with our research. The RA DP patients were older than the RA NDP, this finding is in agreement with the TCM theory that Qi, Xue, Yin, and Yang are more insufficient in older than in younger people (Wu et al., 2012). (Yin, things associated with the physical form of an object; Yang, things associated with energetic qualities; Qi, life force that animates the forms of the world; Xue, dense form of body fluids that have been acted upon and energized by Qi) (Wang et al., 2012). Anti-cyclic citrullinated peptides antibodies levels of patients with RA DP were higher than the RA NDP (Mo et al., 2016), and our experiments show that the level of anti VEGFA165 antibodies in patients with RA DP is lower than that in patients with RA NDP. We also found RA DP patients have higher levels of anti-VEGFA165 antibodies than HC, and confirmed RA have higher levels of anti-VEGFA165 antibodies than HC. Autoantibodies are associated with RA DP, and it may be used to classify TCM pattern.

VEGF is a dimeric glycoprotein, VEGFA, B, C, D and placenta growth factor consisted the VEGF family in mammals. VEGFA included five isoforms: VEGFA121, VEGFA145, VEGFA165, VEGFA189, and VEGFA206 (Olsson et al., 2006), and VEGFA165 was the most abundant factor in most cells and tissues (Neufeld et al., 1996). VEGFA165 can promote the formation of new blood vessels and increased vascular permeability. Inflammation and angiogenesis were interdependent processes, and angiogenesis have significant effects on inflammatory mediators (Oura et al., 2003). VEGF165 has a direct pro-inflammatory effect in the pathogenesis of RA (Yoo et al., 2005). Research has discovered the VEGFA165 levels in sera synovial fluid from RA patients are significantly higher than in synovial fluid from osteoarthritis patients (Lee et al., 2001; Yoo et al., 2005).

**Figure 5:**
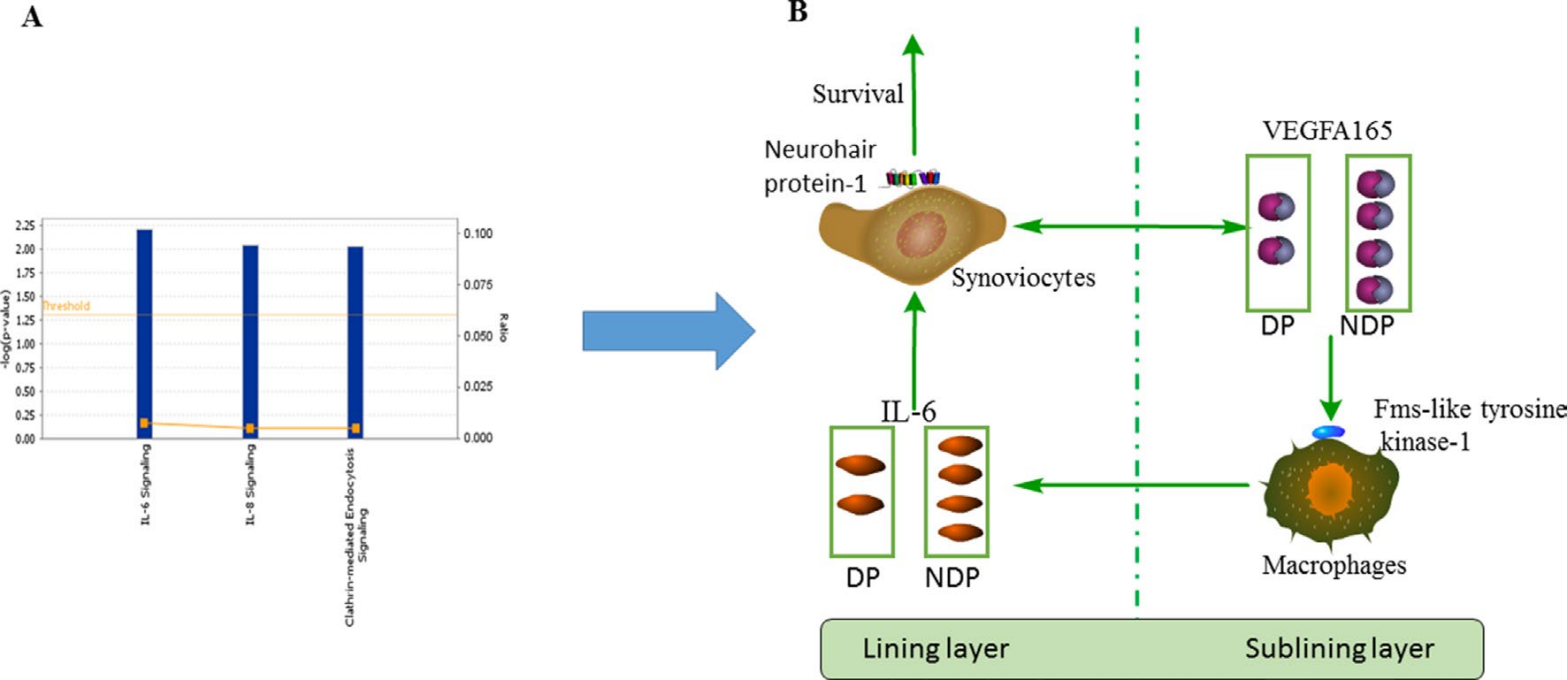
Network analysis and schematic. **(A)** Three pathways related-VEGFA165 protein in RA, included IL-6 signaling, IL-8 signaling and clathrin-mediated endocytosis signling. **(B)** Schematic diagram of the role of VEGFA165 protein in RA.

Our research found there were statistically significant differences in anti-VEGFA165 antibodies between the RA and HC groups. We also found that anti-VEGFA165 antibodies levels of subjects with RA DP were lower than subjects with RA NDP. No statistical differences were found between RA DP and RA NDP groups and other autoantibodies. Through the analysis of the VEGF165-related pathway, we found three pathways related to autoimmunity, including IL-6 signaling, IL-8 signaling and clathrin-mediated endocytosis signaling, the IL-6 signaling pathway is most relevant compared to the other two pathways (**Figure 5A**). VEGFA165 protein is expressed by synovial fibroblasts and synovial macrophages in the synovial tissues of RA patients. A study found that cultured synovial cells are able to secrete VEGFA165 when stimulated with IL-6 (Yoo et al., 2005). The serum levels of IL-6 in RA patients were significantly higher than subjects in HC (Diaz-Torne et al., 2018), and the DP was significantly lower than that in NDP (Peng et al., 2008).VEGFA165 plays a biological role by binding to its receptor subtypes, namely fms-like tyrosine kinase and neurohair protein-1 (Ferrara et al., 2003). VEGFA165 may recruit monocytes around endothelial cells in synovial membranes, where newly employed macrophages can produce IL-6 when stimulated by VEGFA165/fms-like tyrosine kinase-1 binding or *via* cell contact with activated endothelial cells (Yoo et al., 2005). Thus, this creates a self-perpetuating cycle of inflammation. Short interfering RNA down-regulates neurohair protein-1 transcript products and induces spontaneous apoptosis of synovial cells, which is related to the decrease of Bcl-2 expression and the increase of Bax transport to mitochondria (Yoo et al., 2008), which leads to synovial hyperplasia. And in the RA DP, swollen feet joints are more common than in RA NDP (Yuan-Wei and Lou, 2017) (**Figure 5B**). Some of the above findings may partly explain the statistically significant differences in VEGFA165 antibodies between the RA DP and RA NDP groups.

Limitations of this study include the fact that it is the limited sample size with the participants recruited. Due to the fact that RA patients with a single DP are not easy to obtain, since patients usually present with multiple-patterns, larger sample size study is needed in the future.

## Conclusion

In this study, a protein chip containing RA associated autoantigens was successfully constructed. Using this chip, the anti-VEGF 165 antibody was found to be related to the TCM DP of RA, a finding further validated with the ELISA method. This study may promote the development of precise diagnosis and treatment of RA in TCM clinical, provide some clues for understanding the causes of DP of RA.

## Data Availability

The raw data supporting the conclusions of this manuscript will be made available by the authors, without undue reservation, to any qualified researcher.

## Ethics Statement

This study was approved by the Ethics Committee at the Institute of Basic Research in Clinical Medicine, China Academy of Chinese Medical Sciences, and was conducted according to the standards of the Declaration of Helsinki. Written informed consent was obtained from the participants.

## Author Contributions

CL, PC, HZ, AL, JY, and YZ conceived the project. PC, HZ, and CL designed the experiments. HG collected the sera. HZ, PC, and YZ carried out the research. HZ conducted the data analysis and wrote the paper. HZ, YZ, BL, LL, LZ, MB, HG, HX, HF, LX, WY, JY, PC, CL and AL have read and approved the final manuscript.

## Funding

This study was supported by grants from the Fundamental Research Funds for the Central public welfare research institutes (ZZ11-113, ZZ11-056) and National Natural Science Foundation of China (grant no. 81403209).

## Conflict of Interest Statement

The authors declare that the research was conducted in the absence of any commercial or financial relationships that could be construed as a potential conflict of interest.

## Abbreviations

RA, Rheumatoid arthritis; TCM, Traditional Chinese medicine; DP, Deficiency pattern; HC, Healthy controls; VEGF 165, Vascular endothelial growth factor 165; ELISA, Enzyme-linked immunosorbent assay; IL-6, Interleukin 6; IgG, Immunoglobulin G; PBS, Phosphate buffered saline; PBST, Phosphate buffered saline with Tween 20; Cy3, Cyanine dye 3; ESR, Erythrocyte sedimentation rate; CRP, C reactive protein; RF-IgG, Rheumatoid factor immunoglobulin G; RF-IgA, Rheumatoid factor immunoglobulin A; RF IgM, Rheumatoid factor immunoglobulin M; Anti-dsDNA, Anti-distrand-DNA antibody; Anti-CCP, Anti-cyclic citrullinated peptide.

## References

[B1] Arnett F. C. Edworthy S. M. Bloch D. A. McShane D. J. Fries J. F. Cooper N. S. (1988). The american rheumatism association 1987 revised criteria for the classification of rheumatoid arthritis. Arthritis Rheum. 31 (3), 315–324. 10.1002/art.1780310302 3358796

[B2] Boonen A. Severens J. L. (2011). The burden of illness of rheumatoid arthritis. Clin. Rheumatol. 30 Suppl 1, S3–8. 10.1007/s10067-010-1634-9 21359507

[B3] Chai C. Kou J. Zhu D. Yan Y. Yu B. (2011). Mice exposed to chronic intermittent hypoxia simulate clinical features of deficiency of both qi and yin syndrome in traditional chinese medicine. Evid. Based Complement. Alternat. Med. 2011, 356252. 10.1093/ecam/nep226 20047893 PMC3136371

[B4] Dai R. Q. Pan L. Yang L. J. (2016). Correlation of autoantibodies and TCM syndrome differentiation in the patients of systemic lupus erythematosus. World J. Integr. Tradit. West. Med. 11 (6), 808–811. 10.13935/j.cnki.sjzx.160618

[B5] de Hair M. J. Landewe R. B. de Sande M. G. van Schaardenburg D. van Baarsen L. G. Gerlag D. M. (2013). Smoking and overweight determine the likelihood of developing rheumatoid arthritis. Ann. Rheum. Dis. 72 (10), 1654–1658. 10.1136/annrheumdis-2012-202254 23104761 PMC3786633

[B6] Diaz-Torne C. Ortiz M. D. A. Moya P. Hernandez M. V. Reina D. Castellvi I. (2018). The combination of IL-6 and its soluble receptor is associated with the response of rheumatoid arthritis patients to tocilizumab. Semin. Arthritis Rheum. 47 (6), 757–764. 10.1016/j.semarthrit.2017.10.022 29157669

[B7] Ferrara N. Gerber H. P. LeCouter J. (2003). The biology of VEGF and its receptors. Nat. Med. 9 (6), 669–676. 10.1038/nm0603-669 12778165

[B8] Goldbach-Mansky R. Wilson M. Fleischmann R. Olsen N. Silverfield J. Kempf P. (2009). Comparison of Tripterygium wilfordii Hook F versus sulfasalazine in the treatment of rheumatoid arthritis: a randomized trial. Ann. Intern Med. 151 (4), 229–240, w249–251. 10.7326/0003-4819-151-4-200908180-00005 19687490 PMC2938780

[B9] Gu Y. Lu C. Zha Q. Kong H. Lu X. Lu A. (2012). Plasma metabonomics study of rheumatoid arthritis and its Chinese medicine subtypes by using liquid chromatography and gas chromatography coupled with mass spectrometry. Mol. Biosyst. 8 (5), 1535–1543. 10.1039/c2mb25022e 22419152

[B10] Guo H. Niu X. Gu Y. Lu C. Xiao C. Yue K. (2016). Differential amino acid, carbohydrate and lipid metabolism perpetuations involved in a subtype of rheumatoid arthritis with chinese medicine cold pattern. Int. J. Mol. Sci. 17 (10), 1757. 10.3390/ijms17101757 PMC5085781 27775663

[B11] Guo L. K. Luo S. Z. Liao Q. H. Lai R. G. Liu X. L. Liu L. J. (2013). Correlation study of auto-immune antibodies and rheumatoid arthritis patients of Shen deficiency syndrome. Zhongguo Zhong Xi Yi Jie He Za Zhi 33 (5), 619–622. 10.7661/CJIM.2013.05.0619 23905379

[B12] He Y. Lu A. Zha Y. Tsang I. (2008). Differential effect on symptoms treated with traditional Chinese medicine and western combination therapy in RA patients. Complement. Ther. Med. 16 (4), 206–211. 10.1016/j.ctim.2007.08.005 18638711

[B13] Hueber W. Kidd B. A. Tomooka B. H. Lee B. J. Bruce B. Fries J. F. (2005). Antigen microarray profiling of autoantibodies in rheumatoid arthritis. Arthritis Rheum. 52 (9), 2645–2655. 10.1002/art.21269 16142722

[B14] Jiang W. Y. (2005). Therapeutic wisdom in traditional Chinese medicine: a perspective from modern science. Discov Med. 5 (29), 455–461. 10.1016/j.tips.2005.09.006 20704842

[B15] Lee S. S. Joo Y. S. Kim W. U. Min D. J. Min J. K. Park S. H. (2001). Vascular endothelial growth factor levels in the serum and synovial fluid of patients with rheumatoid arthritis. Clin Exp. Rheumatol. 19 (3), 321–324. 10.1002/1529-0131(200105)44:5<1229::AID-ANR209>3.0.CO;2-E 11407088

[B16] Li S. (2002). Advances in TCM symptomatology of rheumatoid arthritis. J. Tradit. Chin. Med. 22 (2), 137–142. 10.3969/j.issn.0255-2922.2002.02.018 12125492

[B17] Liu B. (2014). AB60. Transl. Androl. Urol. 3 (Suppl 1), AB60. 10.3978/j.issn.2223-4683.2014.s060

[B18] Lu C. Niu X. Xiao C. Chen G. Zha Q. Guo H. (2012). Network-based gene expression biomarkers for cold and heat patterns of rheumatoid arthritis in traditional chinese medicine. Evid. Based Complement. Alternat. Med. 2012, 203043. 10.1155/2012/203043 22536280 PMC3318903

[B19] Mo N. Lai R. Luo S. Xie J. Wang X. Liu L. (2016). A Transmembrane Polymorphism of Fcgamma Receptor IIb Is associated with kidney deficiency syndrome in rheumatoid arthritis. Evid. Based Complement. Alternat. Med. 2016, 3214657. 10.1155/2016/3214657 27051449 PMC4802036

[B20] Neufeld G. Cohen T. Gitay-Goren H. Poltorak Z. Tessler S. Sharon R. (1996). Similarities and differences between the vascular endothelial growth factor (VEGF) splice variants. Cancer Metastasis Rev. 15 (2), 153–158. 10.1007/BF00437467 8842486

[B21] Okada T. Tsukano H. Endo M. Tabata M. Miyata K. Kadomatsu T. (2010). Synoviocyte-derived angiopoietin-like protein 2 contributes to synovial chronic inflammation in rheumatoid arthritis. Am. J. Pathol. 176 (5), 2309–2319. 10.2353/ajpath.2010.090865 20304962 PMC2861096

[B22] Olsson A. K. Dimberg A. Kreuger J. Claesson-Welsh L. (2006). VEGF receptor signalling - in control of vascular function. Nat. Rev. Mol. Cell Biol. 7 (5), 359–371. 10.1038/nrm1911 16633338

[B23] Oura H. Bertoncini J. Velasco P. Brown L. F. Carmeliet P. Detmar M. (2003). A critical role of placental growth factor in the induction of inflammation and edema formation. Blood 101 (2), 560–567. 10.1182/blood-2002-05-1516 12393422

[B24] Peng Y. L. Ren Y. G. Zhuang J. H. Ding H. M. Pan W. Y., and Deng, Z.Z.J.J.o.P.M. (2008). Relationship between syndrome patterns of rheumatoid arthritis and interleukin-6 and interleukin-18 levels in serum. J. Prac. Med. 24 (12), 2061–2063. http://en.cnki.com.cn/Article_en/CJFDTotal-SYYZ200812023.htm

[B25] Qiu J. J. Liu Z. Zhao P. Wang X. J. Li Y. C. Sui H. (2017). Gut microbial diversity analysis using Illumina sequencing for functional dyspepsia with liver depression-spleen deficiency syndrome and the interventional Xiaoyaosan in a rat model. World. J. Gastroenterol. 23 (5), 810–816. 10.3748/wjg.v23.i5.810 28223725 PMC5296197

[B26] Sorgjerd E. P. (2018). Type 1 diabetes-related autoantibodies in different forms of diabetes. Curr. Diabetes Rev. 15 (3), 199–204. 10.2174/1573399814666180730105351 30058495

[B27] Tao F. Lu P. Xu C. Zheng M. Liu W. Shen M. (2017). Metabolomics analysis for defining serum biochemical markers in colorectal cancer patients with qi deficiency syndrome or yin deficiency syndrome. Evid. Based Complement. Alternat. Med. 2017, 7382752. 10.1155/2017/7382752 28811829 PMC5546053

[B28] Uhlig T. Moe R. H. Kvien T. K. J. P. (2014). The burden of disease in rheumatoid arthritis. Pharmacoeconomics 32 (3), 841–851. 10.1007/s40273-014-0174-6 24859304

[B29] van Boekel M. A. Vossenaar E. R. van den Hoogen F. H. van Venrooij W. J. (2002). Autoantibody systems in rheumatoid arthritis: specificity, sensitivity and diagnostic value. Arthritis Res. 4 (2), 87–93. 10.1186/ar395 11879544 PMC128920

[B30] Wang L. M. Zhao X. Wu X. L. Li Y. Yi D. H. Cui H. T. (2012). Diagnosis analysis of 4 TCM patterns in suboptimal health status: a structural equation modelling approach. Evid. Based Complement. Alternat. Med. 2012(3), 970985. 10.1155/2012/970985 22550544 PMC3329144

[B31] Wang M. Chen G. Lu C. Xiao C. Li L. Niu X. (2013). Rheumatoid arthritis with deficiency pattern in traditional chinese medicine shows correlation with cold and hot patterns in gene expression profiles. Evid. Based Complement. Alternat. Med. 2013, 248650. 10.1155/2013/248650 24174973 PMC3794642

[B32] Wu T. Yang M. Wei H. F. He S. H. Wang S. C. Ji G. (2012). Application of metabolomics in traditional chinese medicine differentiation of deficiency and excess syndromes in patients with diabetes mellitus. Evid. Based Complement. Alternat. Med. 2012, 968083. 10.1155/2012/968083 22778781 PMC3384925

[B33] Xue D. H. Liu Y. Q. Cai Q. Liang K. Zheng B. Y. Li F. X. (2018). Comparison of Bran-Processed and Crude Atractylodes Lancea Effects on Spleen Deficiency Syndrome in Rats. Pharmacogn. Mag. 14 (54), 214–219. 10.4103/pm.pm_126_17 29720834 PMC5909318

[B34] Yoo S. A. Bae D. G. Ryoo J. W. Kim H. R. Park G. S. Cho C. S. (2005). Arginine-rich anti-vascular endothelial growth factor (anti-VEGF) hexapeptide inhibits collagen-induced arthritis and VEGF-stimulated productions of TNF-alpha and IL-6 by human monocytes. J. Immunol. 174 (9), 5846–5855. 10.4049/jimmunol.174.9.5846 15843589

[B35] Yoo S. A. Kwok S. K. Kim W. U. (2008). Proinflammatory role of vascular endothelial growth factor in the pathogenesis of rheumatoid arthritis: prospects for therapeutic intervention. Mediators Inflamm. 2008, 129873. 10.1155/2008/129873 19223981 PMC2638142

[B36] Yuan-Wei L. I. Lou Y. Q. (2017). Clinical study on the similarities and differences of TCM syndromes of rheumatoid arthritis with and without swollen foot joint. Rheum. Arthritis. 6 (4), 17–21. http://en.cnki.com.cn/Article_en/CJFDTotal-FSBG201708006.htm

[B37] Zhang C. Jiang M. Chen G., and Lu, A.J.E.J.o.I.M. (2012). Incorporation of traditional Chinese medicine pattern diagnosis in the management of rheumatoid arthritis. Eur. J. Integr. Med. 4 (3), e245–e254. 10.1016/j.eujim.2012.02.004

[B38] Zhang W. C. Zhao F. R. Chen J. Chen W. X. (2014). Meta-analysis: diagnostic accuracy of antinuclear antibodies, smooth muscle antibodies and antibodies to a soluble liver antigen/liver pancreas in autoimmune hepatitis. PLoS One 9 (3), e92267. 10.1371/journal.pone.0092267 24651126 PMC3961308

[B39] Zhao W. X. Cui N. Jiang H. Q. Ji X. M. Han X. C. Han B. B. (2017). Effects of radix astragali and its split components on gene expression profiles related to water metabolism in Rats with the Dampness Stagnancy due to Spleen Deficiency Syndrome. Evid. Based Complement. Alternat. Med. 2017, 4946031. 10.1155/2017/4946031 28607573 PMC5457750

[B40] Zhou X. Zhou Z. Jin M. Wang H. Wu M. Song Y. (2004). Clinical study of qingluo tongbi granules in treating 63 patients with rheumatoid arthritis of the type of yin-deficiency and heat in collaterals. J. Tradit. Chin. Med. 24 (2), 83–87. 10.3969/j.issn.0255-2922.2004.02.001 15270253

